# Extrusion-based 3D printing of osteoinductive scaffolds with a spongiosa-inspired structure

**DOI:** 10.3389/fbioe.2023.1268049

**Published:** 2023-09-18

**Authors:** Julie Kühl, Stanislav Gorb, Matthias Kern, Tim Klüter, Sebastian Kühl, Andreas Seekamp, Sabine Fuchs

**Affiliations:** ^1^ Experimental Trauma Surgery, Department of Orthopedics and Trauma Surgery, University Medical Center, Kiel, Germany; ^2^ Department of Functional Morphology and Biomechanics, Kiel University, Kiel, Germany; ^3^ Department of Prosthodontics, Propaedeutics and Dental Material, University Medical Center, Kiel, Germany; ^4^ Department of Electrical and Information Engineering, Kiel University, Kiel, Germany

**Keywords:** critical-sized bone defect, bone implant, extrusion-based printing, 3D printing, scaffold, PCL, calcium phosphate, osteogenic differentiation

## Abstract

Critical-sized bone defects resulting from trauma, inflammation, and tumor resections are individual in their size and shape. Implants for the treatment of such defects have to consider biomechanical and biomedical factors, as well as the individual conditions within the implantation site. In this context, 3D printing technologies offer new possibilities to design and produce patient-specific implants reflecting the outer shape and internal structure of the replaced bone tissue. The selection or modification of materials used in 3D printing enables the adaption of the implant, by enhancing the osteoinductive or biomechanical properties. In this study, scaffolds with bone spongiosa-inspired structure for extrusion-based 3D printing were generated. The computer aided design process resulted in an up scaled and simplified version of the bone spongiosa. To enhance the osteoinductive properties of the 3D printed construct, polycaprolactone (PCL) was combined with 20% (wt) calcium phosphate nano powder (CaP). The implants were designed in form of a ring structure and revealed an irregular and interconnected porous structure with a calculated porosity of 35.2% and a compression strength within the range of the natural cancellous bone. The implants were assessed in terms of biocompatibility and osteoinductivity using the osteosarcoma cell line MG63 and patient-derived mesenchymal stem cells in selected experiments. Cell growth and differentiation over 14 days were monitored using confocal laser scanning microscopy, scanning electron microscopy, deoxyribonucleic acid (DNA) quantification, gene expression analysis, and quantitative assessment of calcification. MG63 cells and human mesenchymal stem cells (hMSC) adhered to the printed implants and revealed a typical elongated morphology as indicated by microscopy. Using DNA quantification, no differences for PCL or PCL-CaP in the initial adhesion of MG63 cells were observed, while the PCL-based scaffolds favored cell proliferation in the early phases of culture up to 7 days. In contrast, on PCL-CaP, cell proliferation for MG63 cells was not evident, while data from PCR and the levels of calcification, or alkaline phosphatase activity, indicated osteogenic differentiation within the PCL-CaP constructs over time. For hMSC, the highest levels in the total calcium content were observed for the PCL-CaP constructs, thus underlining the osteoinductive properties.

## 1 Introduction

Critical-sized bone defects resulting from trauma, inflammation, or tumor resection (Wang et al., 2020) are not able to heal without surgical intervention (Maruyama et al., 2020; Naujokat et al., 2020). Often, these defects require the use of bone grafts, providing mechanical stability and support for the bone repair process. Bone grafts often derive from the patient’s own body (autograft), a donor source (allograft), or are manufactured from synthetic materials (Szpalski and Gunzburg, 2002; Calori et al., 2011; Fillingham and Jacobs, 2016). Autografts contain living cells, growth factors, and native bone structure beneficial for bone repair. However, they are associated with an additional surgical procedure for the patient, which causes additional pain and donor side morbidity (Baldwin et al., 2019). In addition, the bone material for autografts is often limited or insufficient in size, shape, or quality, thus creating a need for alternative sources for bone implants.

New technologies aim to provide synthetic implants, which are manufactured using 3D printing technologies, for instance. The implant can be designed using 3D computer-aided modeling (Leal et al., 2018) processes to adjust the geometry in accordance with the individual shape of the bone defect. In these processes, medical imaging data obtained from 3D scanning (Selli et al., 2020; Zimmerling et al., 2021a; Oberdiek et al., 2021; Yang et al., 2021; Yue et al., 2021) is processed to model the shape of the defect. Besides the outer shape, the internal pore structure needs to ensure both the mechanical stability and the vitality of the tissue. Cancellous bone *in vivo* has a porosity of 40%–95% (Di Luca et al., 2016; Morgan et al., 2018) and is constantly remodeled to cope with external and internal stimuli depending on the physiological conditions.

The pore structure within the implant design should facilitate the ingrowth of new bone tissue and thus improve the integration into the host bone by enabling cell infiltration, nutrient diffusion, and vascularization (Kuboki et al., 2001; Zadpoor, 2014; Kang et al., 2016). All these parameters are essential for the vitality of the bone tissue and its physiological function.

The mechanical stability of the implant is a key prerequisite for load-bearing defects. Thus, the design of the implant as well as the material selection are crucial to achieve adequate mechanical stability (Loh and Choong, 2013; Yang et al., 2021) of a construct.

Due to its high biocompatibility, slow degradation rate, and mechanical stability, one widely used synthetic polymer in bone tissue engineering is polycaprolactone (PCL) (Gregor et al., 2017; Kim et al., 2018; Kobbe et al., 2020a; Zimmerling et al., 2021a). The slow degradation of PCL offers the native bone tissue enough time for remodeling and recreating a natural and functional bone. The degradation products of PCL are considered as non-toxic, and the material has been approved by the Food and Drug Administration (FDA). Preclinical and animal studies have demonstrated the potential of PCL-based scaffolds to promote bone regeneration (Henkel et al., 2013; DeBaun et al., 2019). These studies have evaluated the efficacy of PCL scaffolds in critical-size bone defect models, spinal fusion, and maxillofacial bone reconstruction, showing promising results in terms of tissue integration, neovascularization, and new bone formation (Bose et al., 2012; Dimitriou et al., 2012). In contrast to the promising results, their clinical translation is still in the early stages. Some PCL-based products have received regulatory approval and are being evaluated in clinical trials for bone tissue engineering applications, indicating the growing interest and potential for clinical implementation (Rai et al., 2010; Kobbe et al., 2020a).

Nevertheless, pure PCL has no osteoinductive properties to actively support bone regeneration and osteogenic differentiation of cells. However, osteoinductive properties of PCL can be improved in form of composites using calcium phosphate (CaP), which offers a high bioactivity and similarity to the inorganic part of the bone (Alvarez and Nakajima, 2009; Gin et al., 2018; Saini et al., 2021). Most of the studies so far combine PCL with hydroxyapatite or ß-tricalcium phosphate (Lu et al., 2014; Shim et al., 2017; Eftekhari et al., 2018; Zimmerling et al., 2021a). In contrast, studies with calcium phosphate in form of nanoparticles combined with PCL were not often reported.

CaP materials have the ability to release calcium and phosphate ions into the surrounding environment. These ions are supposed to stimulate cellular activity and act as signaling molecules for osteogenic differentiation (Kolk et al., 2012). In particular, calcium ions play a crucial role in several intracellular signaling pathways involved in osteoblast differentiation and bone mineralization (Jarcho, 1981; Jung et al., 2010; Samavedi et al., 2013; Lei et al., 2015).

Furthermore, composite materials based on CaP and PCL seem suitable to manufacture 3D-shaped implants using 3D printing technologies (Herath et al., 2021). However, the shape of 3D printed scaffolds based on PCL, and CaP, or other printed materials often do not reflect a spongiosa-like structure and resemble simplified grid-like structures (Gómez-Lizárraga et al., 2017; Güney et al., 2018; Ma et al., 2018; Wild et al., 2018; Oberdiek et al., 2021; Sparks et al., 2022). However, this spongiosa-inspired structure, in combination with the components of the bone matrix in the bone tissue, offers a remarkable combination of mechanical strength and porosity.

The aim of this study was to design and to establish the print process for complex 3D scaffolds. The outer and internal structure of the printed constructs was inspired by the structure of the bone spongiosa. Bone spongiosa combines a highly porous structure ensuring the supply of nutrients and a high mechanical stability. In most of the studies, the design of 3D-printed implants does not meet spongiosa-like structures. This structure, however, has unique physiological functions gained via a long-term evolutionary process. The computer aided design process resulted in an up scaled and simplified version of the bone spongiosa also due to the limitations given by the printing technology. Scaffolds were printed using an extrusion-based 3D bioprinter and PCL or PCL-CaP as composite material. The produced implants were characterized in terms of their mechanical properties, measuring the compression strength. Further, the biocompatibility and the influence of the CaP in the composite material were assessed after seeding with MG63 cells, respectively hMSC. For this purpose, we analyzed the impact of CaP in the printed constructs on cell proliferation and osteogenic differentiation.

## 2 Materials and methods

### 2.1 Generation of porous 3D models with python to mimic natural spongiosa of the bone

The rationale for the design of the 3D model was to provide a high porosity within the range of natural bone spongiosa, as well as a adequate mechanical stability. The round geometry of the pores was chosen to support a high interconnectivity. The pores were arranged irregularly in the 3D model to reflect irregularities in the natural bone and to cope with mechanical load, known to influence the bone structure in remodeling processes. In terms of the design process a script to generate a 3D model resembling a spongiosa-like structure was written in Python (version 3.10.8) (R and aybaut, 2019) based on signed distance functions (SDF).

**FIGURE 1:**
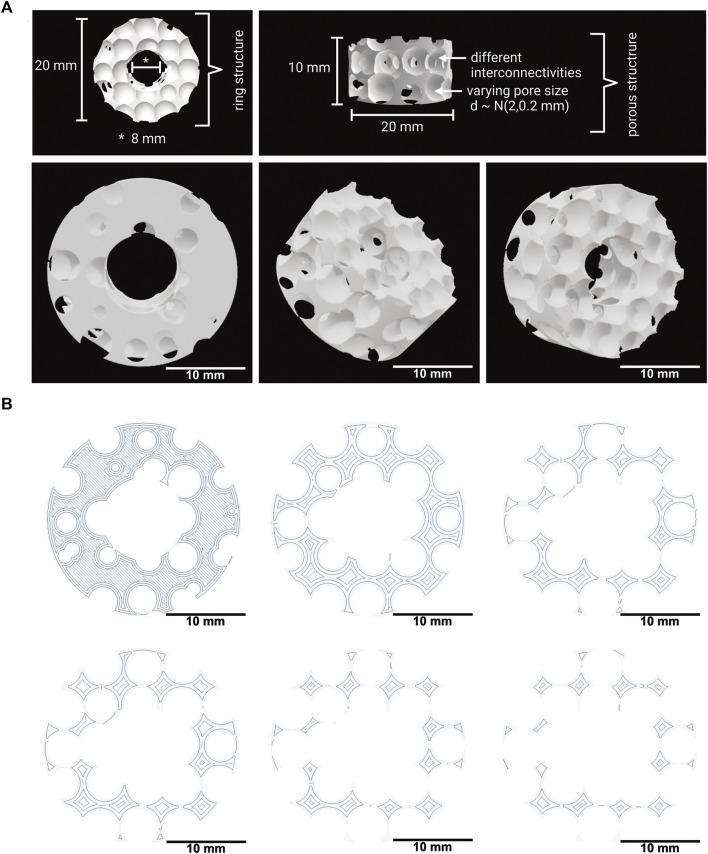
**(A)** 3D model of the implant with a spongiosa-inspired structure. Different views and angles of the spongiosa-inspired 3D model. The 3D model is shaped cylindrically with 10 mm radius and 10 mm height. The pores with different interconnectivities have a size of 2 mm ± 0.2 mm. Scale bar: 10 mm. **(B)** Few slices through the 3D model in different heights. The slices show the structure inside the 3D model. Scale bar: 10 mm.

The 3D model was created based on a cylinder with a radius of 10 mm and a height of 10 mm. This cylinder was modified by subtracting, a cylindrical central cutout with a diameter of 8 mm, resulting in the ring-shaped implant model depicted in Figure 1.

The porosity within the implant model was created by subtracting spheres with a diameter $d∼N\left(2,0.2\text{ mm}\right)$ placed on an equidistant three dimensional grid with a 4 mm spacing. To achieve different interconnectivites, the center of each sphere was randomly offset by $o∼N\left(0,0.2\;mm\cdot I_{3}\right)\in R^{3}$ and every second layer shifted by ${\left(\begin{array}{ccc} 1\;mm & 1\;mm & 0\;mm \end{array}\right)}^{⊤}\in R^{3}$ .

The 3D model was converted into a mesh using the marching cubes algorithm with $2^{22}$ samples and saved in a standard tessellation language (stl) file.The full Python script of the 3D model can be found in the appendix (Python script of the 3D model).

### 2.2 Calculation of the volume, surface, and base area of the 3D model

The overall volume of the 3D implant model, as well as the overall surface area and base area, was calculated using blender (version 2.93.4) (Hendriyani and Amrizal, 2019) and the add-on “3D print toolbox.” For reference, a solid 3D model without the pores but with the same overall geometry of the implant model was created, and volume, surface, and areas were calculated in the same way.

In addition, the porous 3D implant model was sliced in 100 sections in 0.1 mm steps, and the surface of the individual sections was calculated to define the minimal, mean, and maximum areas for the mechanical evaluation (see Section 2.5).

### 2.3 Preparation of PCL calcium phosphate composites for 3D printing

To prepare composite materials for the printing process, PCL with an average molecular weight of 80 kDa in form of 3 mm diameter pellets (Sigma-Aldrich, St. Louis, MO, USA) was combined with CaP nano powder with a particle size less than 150 nm (Sigma-Aldrich). The two materials were mixed in a ratio of 80% PCL and 20% CaP (PCL-CaP) (w/w) under heating at 160 C for 60 min and constant stirring. Using this method, a maximum ratio of 30%, CaP was mixable with PCL, higher ratios of the nanoparticles were not absorbed by the melted PCL. For the print process, we focused on a ratio of 80% PCL and 20% CaP due to the more consistent material properties. From the resulting material, a filament with a diameter of about 5 mm was formed and then cut into pellets with an average diameter of 2–3 mm. The commercially based PCL pellets were used as provided by the supplier.

### 2.4 Extrusion-based 3D printing of bone-like scaffolds

Scaffolds based on PCL or PCL-CaP were printed using BIO X6 (CELLINK, Gothenburg, Sweden), equipped with a thermoplastic printhead and HeartOs (version 2.2.2) and DNA studio (version 3.2.2) software. Using the DNA studio software, the 3D implant model was first sliced into a G-code file with a layer height of 0.41 mm determined by the nozzle size used for the printing process. The infill pattern was set to rectilinear and 99.99%, so that the scaffold reflected the developed 3D model. The established printing settings for the BIO X6 used in this study are shown in Table 1. All scaffolds used in this study were produced in the same way.

**TABLE 1:** Printing parameters of the BIO X6 for PCL and PCL-CaP.

Parameter	Setting
Nozzle diameter	0.4 mm
Temperature printhead	130°C–150°C
Temperature printbed	4°C–30°C
Printing pressure	600–700 kPa
Printing velocity	1 mm/s
Infill density	99.99%
Infill pattern	Rectilinear

### 2.5 Compression strength

The compression strength of PCL and PCL-CaP scaffolds was tested with Zwick Z010 (Zwick/Roell, Ulm, Germany) and the program testXpert 2. Previously, this technique was used to test a range of biological and bioinspired materials (Wa et al., 2019; Wang et al., 2019).

The scaffolds were compressed with a speed of 2 mm/min. until 2500 N were reached and a preforce of 5 N was used before the monitoring process started (please compare Supplementary Figure S1) (Ste et al., 2008). The force was calculated in relation to the mean area as described in Section 2.2.

### 2.6 Preparation of the porous scaffolds for the cell seeding

The printed PCL and PCL-CaP scaffolds were exposed to UV light (385 nm) in a UV chamber (Vilber, Eberhardzell, Germany) twice for 30 min, followed by washing with 70% isopropanol for four times and 15 min each, and then stored in 70% isopropanol for 12 h.

Finally, scaffolds were washed four times for 5 min with PBS under constant shaking and then submerged in tissue buffer (medium 199 GlutaMax^TM^ (gibco® by Life Technologies, California, USA), 1% penicillin/streptomycin (pen/strep), 1% fungizone, and 1% ciprobay. Before the cell seeding, the scaffolds were washed again four times with PBS.

BACT/ALERT® SA (Biomeriuex) sterility test was used to confirm the disinfection process and indicated no growth of aerobic microorganisms.

### 2.7 Cell seeding and cultivation of the spongiosa-inspired scaffolds

Biocompatibility and functionality of 3D printed scaffolds were assessed using cell types with an osteoblastic phenotype. For this purpose, the osteosarcoma cell line MG63 was cultivated in Dulbecco’s Modified Eagle Medium (DMEM) (PAN Biotech, Aidenbach, Germany) supplemented with 10% fetal bovine serum (FBS) (PAN Biotech, Aidenbach, Germany), 1% L-Glutamine (gibco® by Life Technologies, California, USA) and 1% pen/strep (Biochrom, Berlin, Germany) in the incubator at 37°C and 5% CO_2_. MG63 cells from different passages were used for the experiments. After detaching the cells using standard procedures, the scaffolds were seeded with a density of 5 × 10^6^ cells per scaffolds in ibidi dishes (Ibidi GmbH FCA & Gräfelfing, Germany). The sealed dishes were mounted on a rotating heating oven in reaction tubes. After 24 h at 37°C, the seeded scaffolds were further cultivated in fresh phenol red free DMEM. The medium was changed every third day.

In addition, selected experiments were performed with human mesenchymal stem cells (hMSC) to monitor the impact of the implants on patient-derived cells. For this purpose, hMSC were isolated from femoral heads of adult patients undergoing total hip replacement surgery as described before (Kolbe et al., 2011; Wang et al., 2021). The use of human tissue was permitted by the local ethical advisory board of the university medical center in Kiel (Approval number - D459/13) and included the consent of the individual donors.

hMSC were cultivated in osteogenic differentiation medium ((DMEM/F12) (PAN Biotech, Aidenbach, Germany), 10% FBS, 1% pen/strep, 50 mM ascorbic acid (Sigma-Aldrich), 10 mM ß-glycerol phosphate (Sigma-Aldrich), 0.1 mM dexamethasone (Sigma-Aldrich).

Experiments with hMSC were conducted with cells in passage numbers 3 and 4, and experiments were performed using cells from different donors using the same settings as described for the MG63.

### 2.8 Confocal laser scanning microscopy of 3D printed constructs seeded with cells

PCL and PCL-CaP implants seeded with MG63 cells or hMSC were fixed after the indicated time points of cultivation (1, 7 or 14 days) using 4% paraformaldehyde (PFA) (Affymetrix, Cleveland, USA) and 3% glutaraldehyde (GA) (Sigma-Aldrich) for 30 min.

After fixation, the samples were washed three times for 10 min with PBS followed by permeabilization using 0.5% Triton^TM^ X-100 (Sigma-Aldrich) for 30 min, and washed again with PBS.

To block unspecific binding sites, the samples were pretreated with 1% bovine serum albumin (BSA) (Millipore, Kankakee, USA) dissolved in PBS for 30 min, followed by washing steps. Samples were stained using Phalloidin TRITC (Tetramethyl rhodamine) for 45 min, and followed by washing steps. Hoechst 33342 was used for nuclear counterstain. Imaging of the stained samples was performed using a confocal laser scanning microscopy (CLSM) LSM 800 (Zeiss, Oberkochen, Germany).

### 2.9 Scanning electron microscopy

PCL and PCL-CaP scaffolds seeded with MG63 cells were fixed with 4% PFA and 3% GA after 7 and 14 days. After washing three times for 5 min with PBS, the scaffolds were dehydrated in an ascending alcohol series. The scaffolds were incubated at room temperature for 15 min using an alcohol series of 50% 60%, 70%, 80%, 90%, 95% and 100% ethanol. The step with 100% ethanol was repeated three times to replace all water in the sample. Then the ethanol was evaporated under an air vent until the samples were dry.

The dried samples were mounted onto a holder with a carbon Leit-Tab (Plano GmbH, Wetzlar, Germany) and sputter-coated with gold-palladium (10 nm thickness) using a Leica EM SCD 500 High-Vacuum sputter-coater (Leica Microsystems GmbH, Wetzlar, Germany). The samples were imaged using the scanning electron microscope (SEM) Hitachi TM300 (High-Tech., Tokkyo, Japan) and were scanned at 15 kV.

### 2.10 Energy-dispersive x-ray spectroscopy

Energy-dispersive x-ray spectroscopy (EDX) analysis was performed using a Philips XL 30 CP.

SEM (Philips, Amsterdam, Netherlands). Prior to analysis samples were sputtered with gold-palladium with 10 nm thickness. The SEM was operated with 25 kV. Three different areas on scaffolds were chosen so that 2,100 counts per second (CPS) were registered and dead time was 30%–35%. Measurements were performed for a period of 200 live seconds (Lsec) Figure 3.

### 2.11 DNA quantification to asses cell seeding and cell proliferation

To analyze the proliferation rate of MG63 cells seeded onto the different scaffold materials, the DNA content was determined using a Quant-iT PicoGreen dsDNA assay kit (Moelcular probes, Eugene, OR, USA) at day 1, 7 and 14.

The scaffolds consisting of PCL and PCL-CaP were placed in nuclease free water (Nalgene, Thermo Fisher Scientific, Massachusetts, USA), frozen at −80°C and thawed for three cycles. Afterwards scaffolds were additionally sonicated (MSE, Henderson Biomedical, UK) three times. The DNA content was determined in accordance with the manufacturer’s protocol using PicoGreen as a DNA binding fluorescent dye. A standard curve was created using standard lambda DNA solutions. The DNA of each sample was quantified by measuring the fluorescence using a microplate reader, SpectraMax® iD3 (Molecular Devices, California, USA) at an excitation wavelength of 485 nm and an emission wavelength of 535 nm. The samples were applied and measured in technical triplets.

### 2.12 Alkaline phosphatase assay

Medium retrieved from MG63 cell-seeded scaffolds was collected on day 1, 4, 7, 10 and 14. The osteogenic activity was evaluated using an alkaline phosphatase assay kit (abcam, Cambridge, UK) in accordance with the manufacturer’s protocol (Zhang et al., 2021). The absorbance was measured with a microplate reader at 405 nm.

### 2.13 Alizarin red staining and quantification

The mineralization of cell-seeded constructs was analyzed by Alizarin red staining (ARS) using an osteogenesis assay kit (Millipore, Billerica, MA, USA) after 7 and 14 days of culture.

ARS was performed to assess the CaP content of the constructs with MG63 cells as well as the CaP content in the PCL-CaP composite material-based implants without cells. The assay procedure followed the instructions by the manufacturer, and the deposition of calcified matrix on the samples was documented with the 3D microscope Keyence VR-3100 (Keyence Deutschland GmbH, Neu-Isenburg, Germany).

For the quantitative analysis of the mineralization, ARS was extracted with 10% (w/v) cetylpyridium chloride (CPC) for 48 h on an orbital shaker. Alizarin red solutions from stained cell-laden scaffold and standards in a high range (2 mM, 1 mM, 500 mM, 250 mM, 125 mM, 62.5 mM, 31.3 mM, 0 mM) were prepared in ARS dilution buffer and added to a 96-well plate. The absorbance of the samples was measured at a wavelength of 405 nm in a microplate reader for quantification.

### 2.14 RNA isolation and semi-quantitative real time polymerase chain reaction

The RNA was isolated using the peqGOLD total RNA kit (peQlab, VWR, Pennsylvania, USA) in accordance with the manufacturer’s protocol.

The scaffolds seeded with MG63 cells were incubated with 2 mL RNA Lysis Buffer T, and the cell lysates were collected. The lysates of three samples were pooled together for further RNA extraction.

A DNase I treatment was included in the isolation procedure, and the RNA concentration of each sample was measured by a nanodrop 2000c (Thermo Scientific, Langenselbold, Germany) at wavelengths of 260 nm/280 nm. For each sample, 1 µg RNA was transcribed into cDNA using the High-Capacity RNA-to-cDNA^TM^ kit (Applied Biosystems, Vilnius, Lithuania), following the manufacturer’s instructions.

The qPCR was carried out using a total volume of 20 µL for each reaction; 3.2 µL cDNA was mixed with 10 µL SYBR ^TM^ Select Master Mix (Applied Biosystems), 2 µL Quanti-Tect primer assays (Qiagen, Hilden, Germany), and 4.8 µL nuclease-free water. All primers are listed in Table 2.

**TABLE 2:** Quanti-Tect Primer Assays (Qiagen) for qPCR.

Gene	QuantiTect primer assay	Catalog number
Alkaline phosphatase	Hs_ALPL_1_SG	QT00012957
Integrin-ß1	Hs_INGT B1_1_SG	QT00068124
Osteocalcin	Hs_BGLAP_1_SG	OT00232771
Collagen type 1 α 1	Hs_COL1A_1_SG	QT00037793
60S Ribosomal protein L13a	Hs_RPL13A_1_SG	QT00089915

The gene for ribosomal protein 13A (RPL13A) was used as internal housekeeping gene. For the amplification, a two-step PCR program was initiated (50°C for 2 min, 95°C for 2 min, 40 cycles, 95°C for 15 s, and 60°C for 60 s). The relative gene expression was calculated using the ΔΔ Ct method.

### 2.15 Statistics

The data was evaluated with R studio (version 4.2.1) (Team, 2021). Data depicted result from different technical and biological replicates, as specified in the individual chapters and figures.

The statistics were calculated with Welch’s *t*-Test and an ANOVA with a posthoc Tukey. Statistical significance was indicated as *p* < 0.05 (*), *p* < 0.01 (**), *p* < 0.001 (***), and *p* < 0.0001 (****).

## 3 Results

### 3.1 Development and structure of a 3D implant model with a spongiosa-inspired structure

A 3D model for bone implants that mimics the natural architecture of the bone spongiosa was developed based on a script in python, as depicted in Figure 1A. The 3D model aims to resemble a simplified bone spongiosa with scaled-up dimensions in terms of pore sizes and overall geometry of the bone spongiosa. The size of pores within the 3D model is randomly distributed by $N\left(2,0.2\text{ mm}\right)$ . In comparison, the pore size distribution within the natural spongiosa is more heterogeneous, ranging from a few to several hundred micrometers (Murphy and O'Brien, 2010; Abbasi et al., 2020).

The calculated values for the volume, surface area, and base area of the 3D spongiosa model are summarized in Table 3. The calculated volume of the spongiosa 3D model sums up to 929 mm^3^, comprising a 2.8 times lower volume than the solid body with the same dimensions. Due to this lower volume, the scaffold also has a lower weight compared to the solid counterpart.

**TABLE 3:** Table of the calculated volume, surface area and base area for the 3D model.

3D model	Volume [mm^3^]	Surface area [mm^2^]	Base area [mm^2^]	Porosity [%]
Solid ring structure	2,638.93	1,407.43	263.89	0
Spongiosa-inspired structure	928.9	2,264.32	Min.: 5.49	35.2
			Max.: 218.53	
			Mean.: 83.075	

The calculated surface area of the 3D model adds up to 2,264 mm^2^ in total, revealing a 1.6 times larger area than the model without pores. The overall porosity of the scaffold is 35%. These parameters enable a larger surface for cell growth and integration of the implant into the bone tissue. In comparison, the porosity of cancellous bone ranges from 40% to 95% (Di Luca et al., 2016; Morgan et al., 2018).

Further, the model was sliced into different sections, followed by calculation of the area using blender as described in Section 2.2, and resulting in a 3.18 times lower area of the porous than the solid ring model (Figure 1B). The area ranges from 5.49 mm^2^ to 218.53 mm^2^, thus revealing an area difference by a factor of 4.

### 3.2 Established parameters for extrusion-based printing (BIO X6) of the scaffolds in accordance with the 3D-model

Using the BIO X6 printing protocols for the materials PCL and PCL-CaP (80% PCL, 20% CaP), the established parameters to print the scaffolds used in this study are summarized in Table 1. In brief, the materials PCL and PCL-CaP reached the best viscosity for printing using a temperature of 130°C–145°C. Higher temperatures than 130°C–145°C led to changes in the PCL material in color, indicating a loss of chemical stability. The pressure of 600–700 kPa is the maximal pressure the printer can bear and was necessary to cope with the viscosity. The printing speed was established empirically as this resulted in the best optically controlled printing resolution.

### 3.3 Macroscopic structure and mechanical properties of printed scaffolds

**FIGURE 2:**
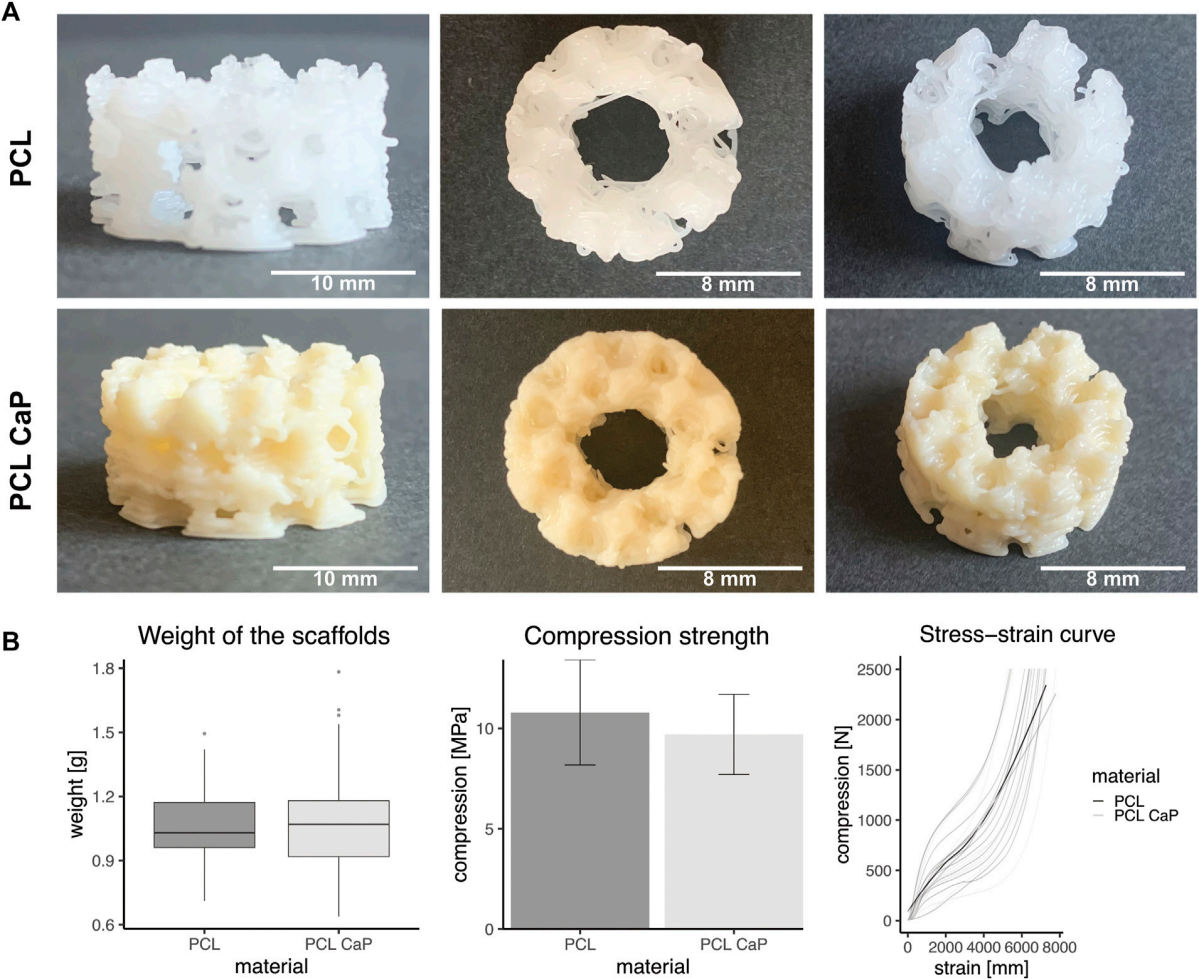
Mechanical properties of the printed scaffolds consisting of PCL and PCL-CaP. **(A)** Different views of the printed constructs. Left: Scale bar: 10 mm, Middle/Right: Scale bar: 8 mm. **(B)** Left: The mean weight of the scaffolds based on PCL and PCL-CaP, *n* = 129, Middle: Compression strength measured with an increasing force up to 2,500 N and a speed of 2 mm/s. The statistics were performed with Welch’s *t*-Test, *n* = 12. Right: Force-distance-curve for both materials of the printed scaffolds. The saturated grey and light grey curves show the mean trend curves of the tested samples, n = 12.

The overall shape and macroscopic structure of the 3D printed constructs are depicted from different angles for both types of materials in Figure 2A. The general appearance of the scaffolds was similar; however, after the addition of the CaP nanoparticles, the polymer became more opaque.

After printing, the weight of the PCL and PCL-CaP scaffolds was determined to monitor potential differences derived from the material composition or the printing processes. However, the weight of the printed scaffolds based on the two materials showed no significant differences (Figure 2B), as indicated by a mean weight for PCL-CaP of 1.08 g ± 0.23 g, which was not significantly higher than the mean weight of the scaffolds consisting of PCL of 1.04 g ± 0.15 g.

The mechanical properties of the printed scaffolds based on PCL and PCL-CaP were investigated by measuring the compression strength, an essential factor for bone implants. When the compression force was normalized to mean area of the scaffold, the compression strength was 10.79 MPa ±4.12 MPa for PCL and 9.7 MPa ±3.15 MPa for PCL-CaP (Figure 2B). The compression strength values for the scaffolds based on PCL-CaP were slightly lower. The mean force-distance curves for PCL and PCL-CaP scaffolds showed similar profiles, as depicted in Figure 2B.

Overall, the CaP nanoparticles showed no negative influence on the compression strength. In summary, the compression strength of the printed constructs independent from the material is in the range of natural bone tissue or comparable to other reported bone implants (Keaveny and Hayes, 1993; Van Der Linden et al., 2001; Weir, 2004).

### 3.4 Element analysis by EDX spectroscopy

**FIGURE 3:**
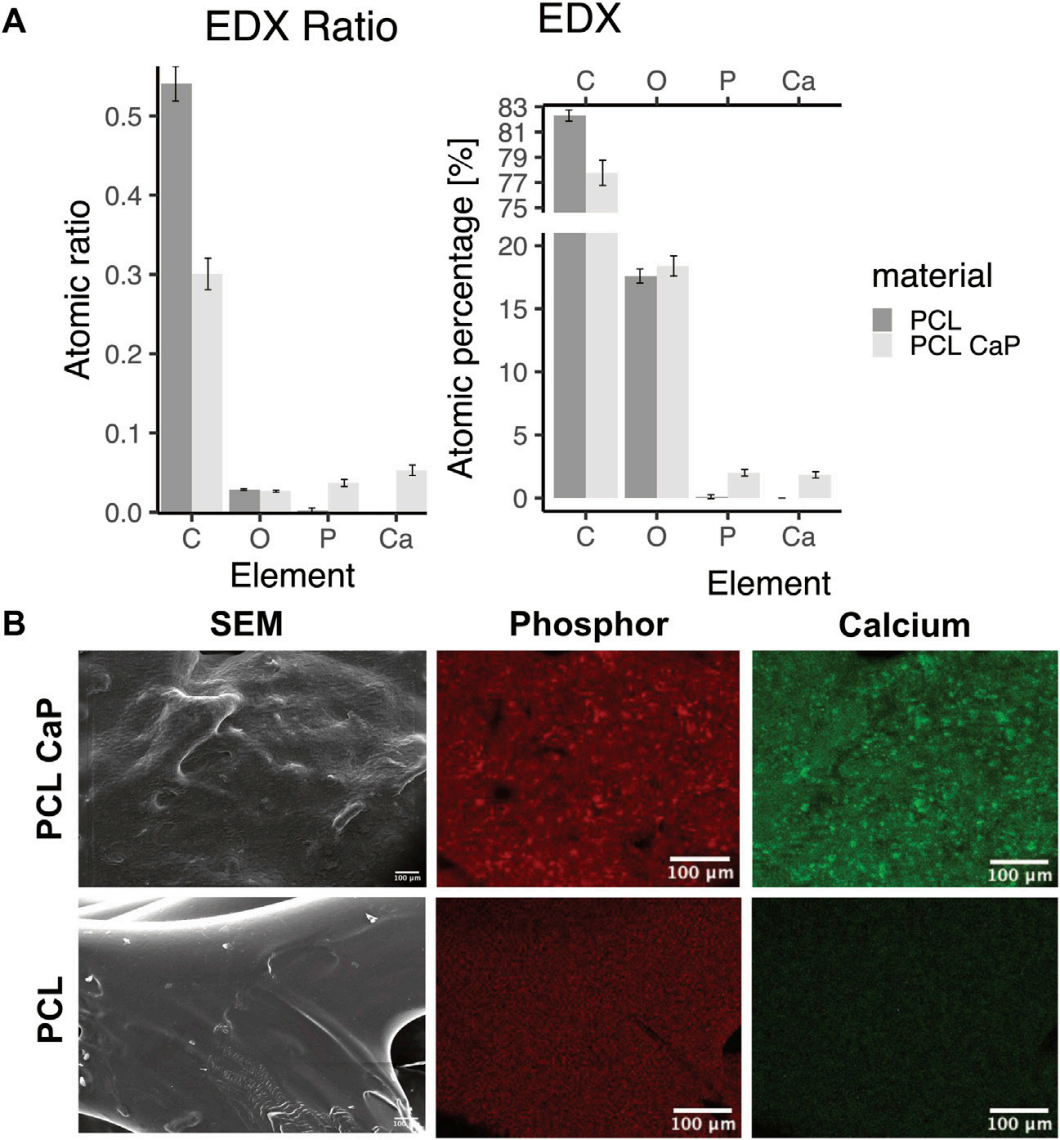
Energy-dispersive x-ray spectroscopy for scaffolds consisting of PCL and PCL-CaP. **(A)** EDX ratio and atomic percentage for carbon (C), oxygen (O), phosphor (P), and calcium (Ca). The EDX analysis was performed for different regions of the scaffolds, *n* = 2 scaffolds. **(B)** Element map for P and Ca for a selected area of the scaffolds, as well as the same region as SEM picture.

Element analysis was performed by EDX spectroscopy to identify calcium and phosphor in the printed scaffolds (Figure 3). For the PCL-CaP scaffolds calcium and phosphor were clearly detectable in contrast to the pure PCL scaffolds (Figure 3A) and the elements were equally distributed on the material surface (Figure 3B).

### 3.5 Morphological evaluation of cell-seeded constructs using confocal and scanning electron microscopy

**FIGURE 4:**
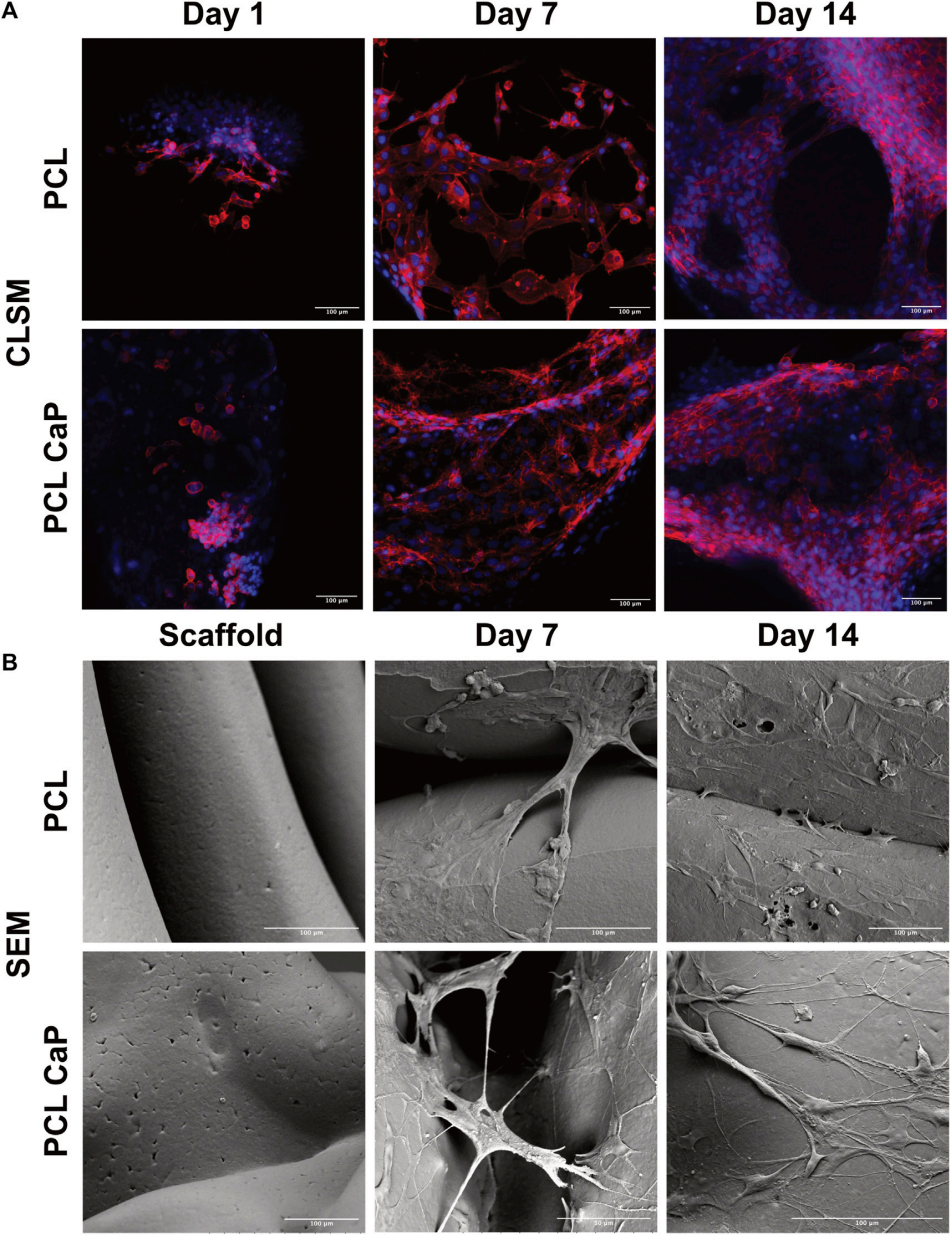
CLSM **(A)** and SEM **(B)** for the MG63 cell-seeded scaffolds. The cells were stained with Phalloidin TRITC (Donzelli et al., 2007) and Hoechst 33342 (blue) to visualize the actin skeleton and the nucleus **(A)**, Scale bar: 100** **µm. **(B)** The surface of the cell-free scaffolds (left) and the cell-laden scaffolds after 7 and 14 days (right), Scale bar: 100** **µm.

3D printed constructs were seeded with MG63 cells to analyze the impact of the printed constructs on cell performance and then subjected to CLSM (Figure 4A) and SEM (Figure 4B) at different time points after cell seeding.

For CLSM, the cells were stained with Phalloidin TRITC to reveal the cellular cytoskeleton, and Hoechst 33342 was used as a nuclear counterstain. The samples from different time points after cell seeding (day 1, 7 and 14) were analyzed to follow cell attachment, viability, seeding density, and overall morphology during the culture process. After 24 h, the MG63 cells showed a rounded morphology for both types of scaffolds (Figure 4A). With progressing culture from day 1 to day 7 (Figure 4A), the actin skeleton of the MG63 cells seemed more elongated, indicating a progressing cell attachment for both types of scaffolds. At day 14, the MG63 cells formed larger cell clusters and stretched out even further in comparison to the earlier time points (Figure 4A). Overall, the cell morphology and attachment of the MG63 cells showed no differences between the two tested materials (PCL and PCL-CaP). However, slight differences in cell density may exist in individual areas, thus quantitative evaluation of cell performance was performed, as indicated in upcoming sections.

**FIGURE 5:**
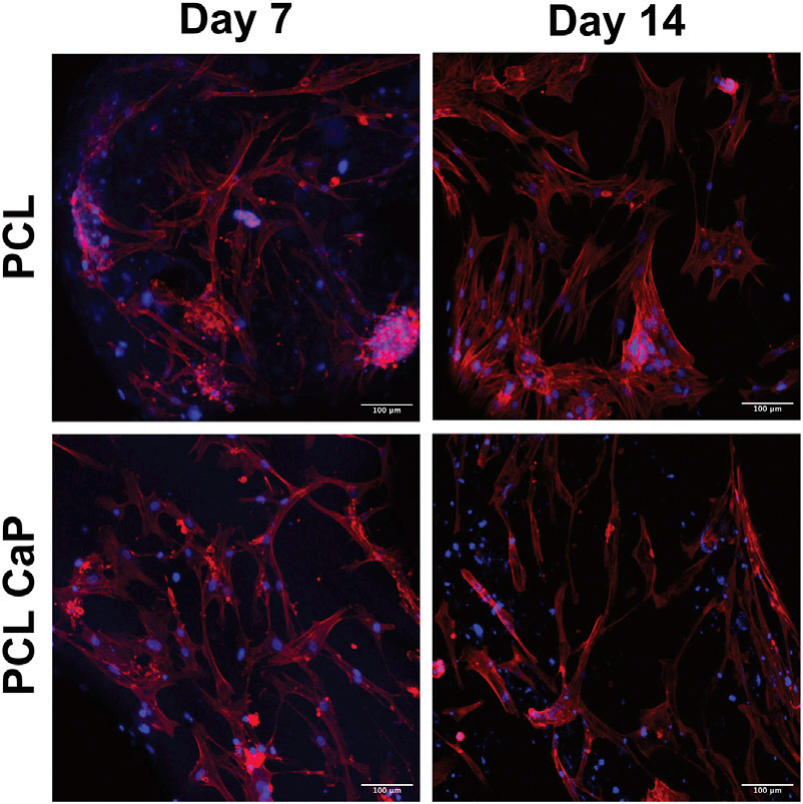
CLSM of hMSC-seeded scaffolds. The hMSC grown on top of the printed scaffolds stained with Phalloidin TRITC (Donzelli et al., 2007) and Hoechst 33342 (blue). Scale bar: 100 µm.

Similar to the CLSM findings, cellular attachment and growth were confirmed by SEM analysis. In contrast to CLSM, SEM allowed the depiction of the implants and their surface structure. The SEM images of the MG63 cell-seeded scaffolds showed that the cells adhered tightly to both types of materials (Figure 5B). The cells used the printed fibers to adhere to the implant surface, but partly span also between the fibers (Figure 5B). For both types of materials, SEM images indicated slight variations regarding the cellular confluency in different areas.

In samples without cells, the surface of PCL seemed slightly smoother compared to the surface in PCL-CaP samples (Figure 4B). On the surface of PCL-CaP indented structures appeared that were not visible for the PCL alone, indicating potential micropores caused by the CaP nanopowder.

Similar to the results for MG63, the actin skeleton of the hMSC showed an elongated structure at day 7. From day 7 to 14, the hMSC build an elongated actin skeleton and coherent cell layers. Overall, hMSC showed the same morphological appearance on the PCL and PCL-CaP scaffolds and the morphologal data indicated a high biocompatibility (Figure 5) of the implants.

### 3.6 Influence of material composition on cellular proliferation

**FIGURE 6:**
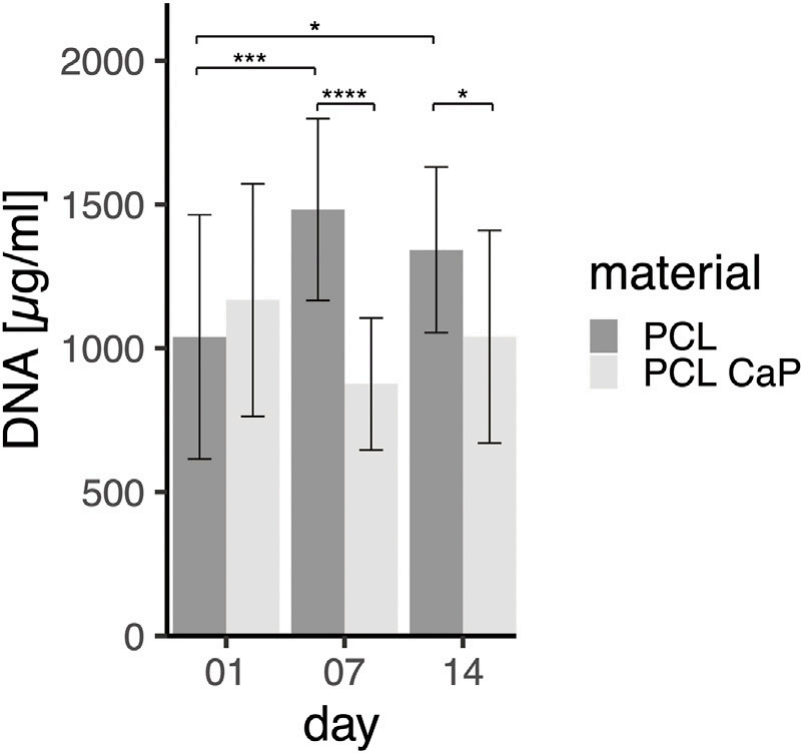
DNA Quantification for MG63 cell-seeded constructs on different time points. The MG63 cells show proliferation from day 1 to day 7 at the material PCL. The statistical evaluation was determined using ANOVA with posthoc Tukey test, *n* = 4 from different passages and 2 technical replicates. Statistical significance level: *p* < 0.05 (*), *p* < 0.001 (***), and *p* < 0.0001 (****).

Despite the promising morphological data as indicated above, quantitative analysis methods are essential to compare the cellular performance of 3D printed implants consisting of PCL or PCL-CaP. Thus, the DNA amount and cellular proliferation of MG63 cells seeded onto the scaffolds were analyzed after 24 h, 7 days, and 14 days of cultivation (Figure 6).

Both types of scaffolds were seeded with the same cell numbers of 5 × 10^6^ MG63 cells per scaffold. As shown by the DNA levels, 24 h after seeding, similar numbers of MG63 cells initially adhered to the different materials. On day 7 and day 14, significantly different DNA concentrations were observed for PCL and PCL-CaP, revealing significantly lower levels of DNA for the PCL-CaP samples.

While levels of DNA for PCL samples significantly increased from day 1 to day 7, indicating cellular proliferation in this time frame, the levels of DNA on day 14 were not further increased but were still significantly higher compared to day 1 for the PCL samples. These data indicated cellular proliferation of MG63 cells from day 1 to day 7 for the PCL-based scaffolds. In comparison, no significant increase in DNA levels was observed during the culture process for PCL-CaP samples, thus revealing no cellular proliferation for these scaffolds.

### 3.7 Evaluation of genes associated with osteogenic differentiation and bone repair by semi-quantitative real-time PCR

**FIGURE 7:**
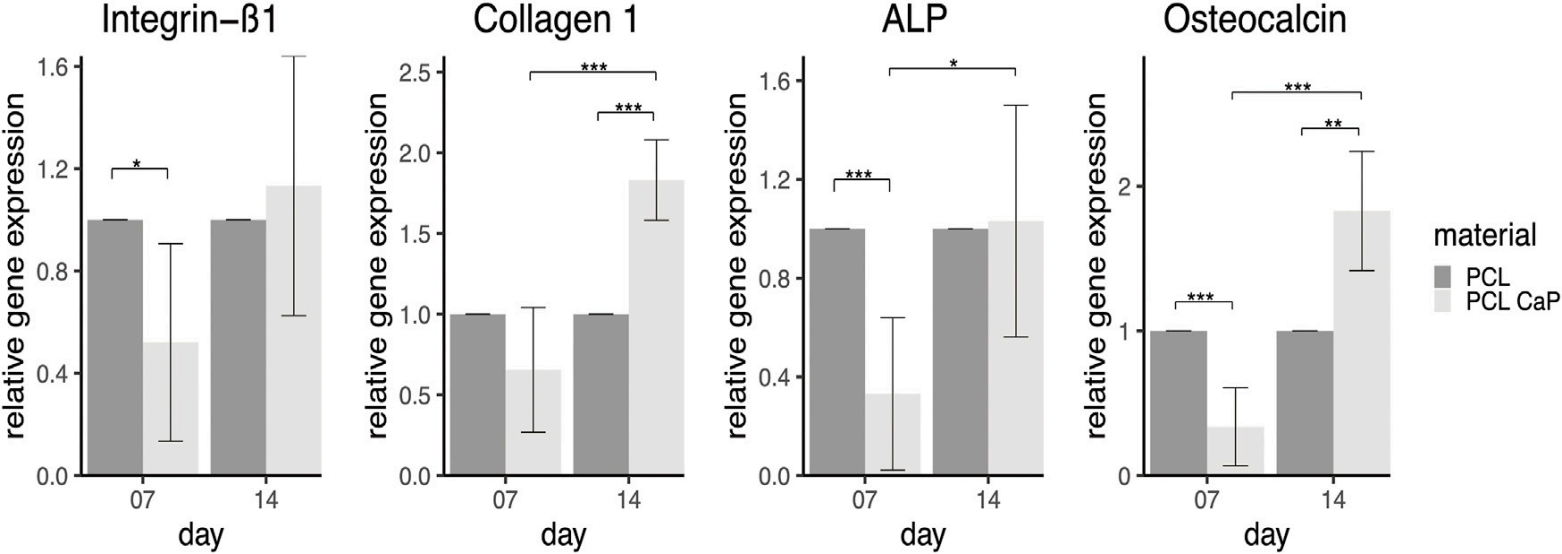
Semi-quantitative gene expression for osteogenesis and adhesion related markers of MG63 cells cultured on printed samples. The statistical evaluation was performed using Welch’s *t*-test, *n* = 3 independent experiments. Statistical significance level: *p* < 0.05 (*), *p* < 0.01 (**), and *p* < 0.001 (***).

Semi-quantitative real-time PCR for cell-seeded constructs consisting of PCL or PCL-CaP was performed to assess the gene expression of several markers associated with cell adhesion and osteogenic differentiation (Figure 7). The gene expression was analyzed after 7 and 14 days of cultivation to gain insight into temporal development of differentiation-associated markers in MG63 (Figure 7).

The adhesion-related gene integrin ß1 in MG63 showed significantly higher gene expression rates for cells grown on the PCL-based implants on day 7 compared to the scaffolds based on PCL-CaP. This might indicate better adhesion of the MG63 cells on the pure PCL scaffolds at the early phase of culture, whereas the integrin ß1 expression and associated cell adhesion tentatively improve for PCL with CaP over time from 7 to 14 days. Two differentiation-associated markers, including collagen type 1, the main structural protein in bone tissue, and osteocalcin involved in the calcification of bone tissue, showed the highest expression levels for PCL combined with CaP at 14 days, revealing significant differences to PCL-based scaffolds at the same time point. The gene expression levels of these markers showed a tentative increase over time, in accordance with their role in the differentiation process. ALP gene expression levels also showed a significant increase from day 7 to day 14 for PCL with CaP derived samples, underlining an osteogenic differentiation process of the cells.

### 3.8 Morphological and quantitative evaluation of calcification processes in 3D printed constructs

Calcification is a functional key feature of osteogenic differentiation and bone repair processes. AR stains the calcium-rich areas, providing a visual indication of mineralization. Thus, AR assays were performed to monitor calcification of the cell-seeded constructs over the culture time. In addition, the assay enables, at least partly, the quantification of the integration of CaP nanoparticles in the PCL and CaP composites. Images of different CaP ratios in the scaffolds are accordingly depicted in Supplementary Figure S2.

**FIGURE 8:**
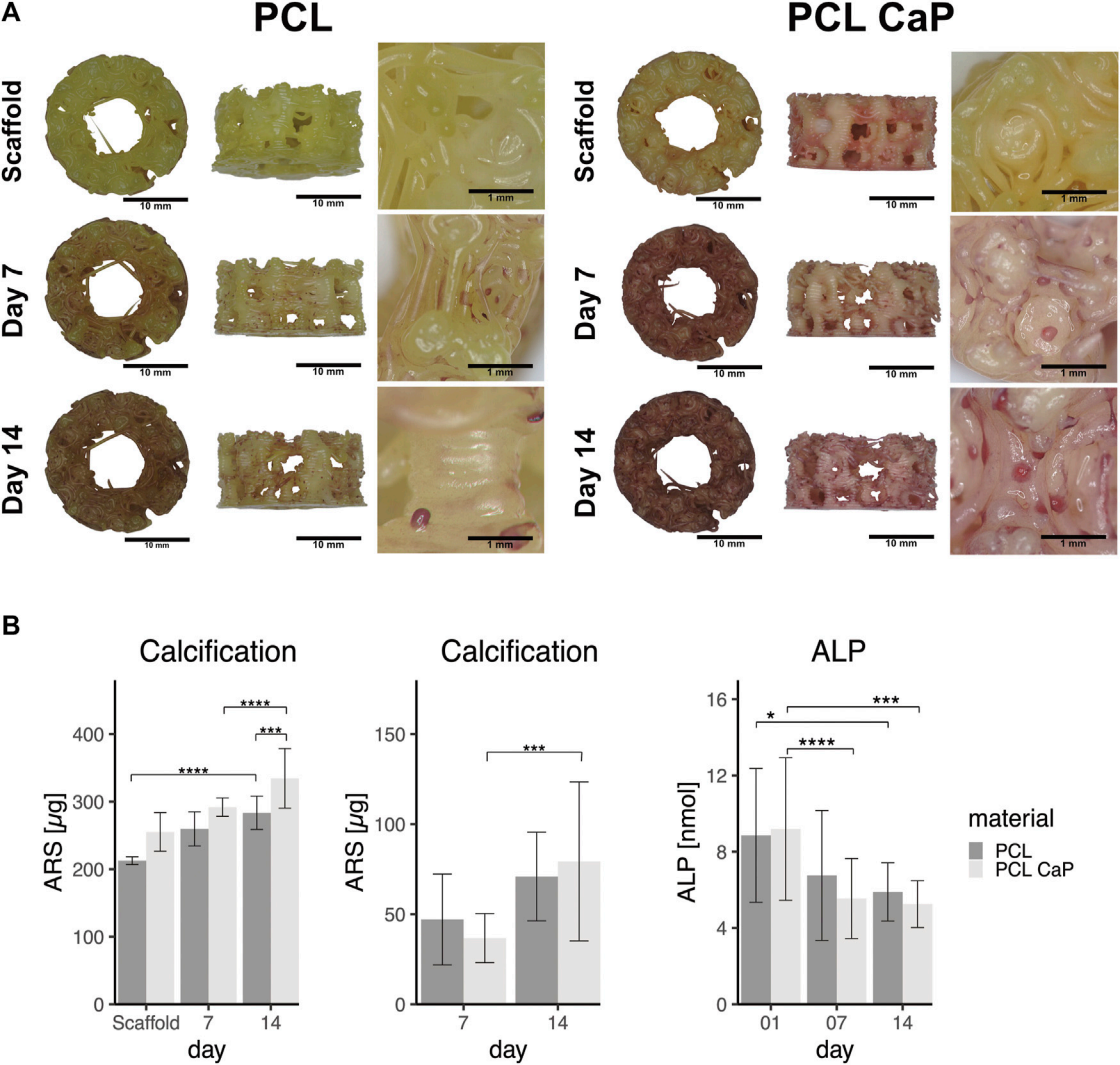
The calcification in MG63 cell-seeded scaffolds using Alizarin Red staining. **(A)** Light microscopy and morphological appearance of calcified areas over culture time. **(B)** Quantification of calcification in MG63 cell-seeded scaffolds. Left: Total values for calcification from scaffolds without cells and MG63 cell-seeded constructs, Middle: Cell-based calcification (scaffold values subtracted). The statistical evaluation was performed using ANOVA with posthoc Tukey, *n* = 3, from three different passages with three technical replicates. ALP activity in cell-seeded construct showed decreasing values over the time. The statistical evaluation was performed using ANOVA with posthoc Tukey with *n* = 6, from six different passages with three technical replicates. Statistical significance level: *p* < 0.05 (*), *p* < 0.001 (***), and *p* < 0.0001 (****).

Morphological assessmenf calcification processes and quantification are depicted in Figures 8A, B for both types of 3D printed scaffolds seeded with MG63 cells. In the light microscopy images, the ARS indicated diffuse background staining for the pure and non-cell-seeded PCL-CaP material. This background was not visible for the pure PCL samples without the cells (Figure 8A), thus indicating the CaP within the composite implants. When the scaffolds were seeded with cells, we observed an increase in alizarin red clusters with time of culture for both materials. However, these clusters were most prominent on the PCL-CaP after 14 days for cell-seeded constructs (compare Figure 8A). These microscopic observations were confirmed by the quantitative assessment of alizarin after extraction and photometric evaluation (Figure 8B). Here we depict both the overall calcification, including the total values from the cell-seeded constructs, as well as the values for the cell-mediated calcification calculated by subtracting the values from the scaffold.

The highest values in terms of calcification were observed for the PCL-CaP samples seeded with cells and cultured for 14 days (Figure 8B left graph). The values for scaffolds based on pure PCL were significantly lower, thus demonstrating a positive impact of the CaP on the calcification process in the 3D printed scaffolds. After the subtraction of the values from the scaffolds, a significant increase in calcification for PCL-CaP over time was observed, indicating cell mediated calcification and osteogenic differentiation. In comparison, the ALP activity used as a marker of early osteogenic differentiation decreased over time, resembling a contrary profile to the AR intensity, which has been reported as typical for the osteogenic differentiation processes (Frank et al., 2002; Donzelli et al., 2007).

**FIGURE 9:**
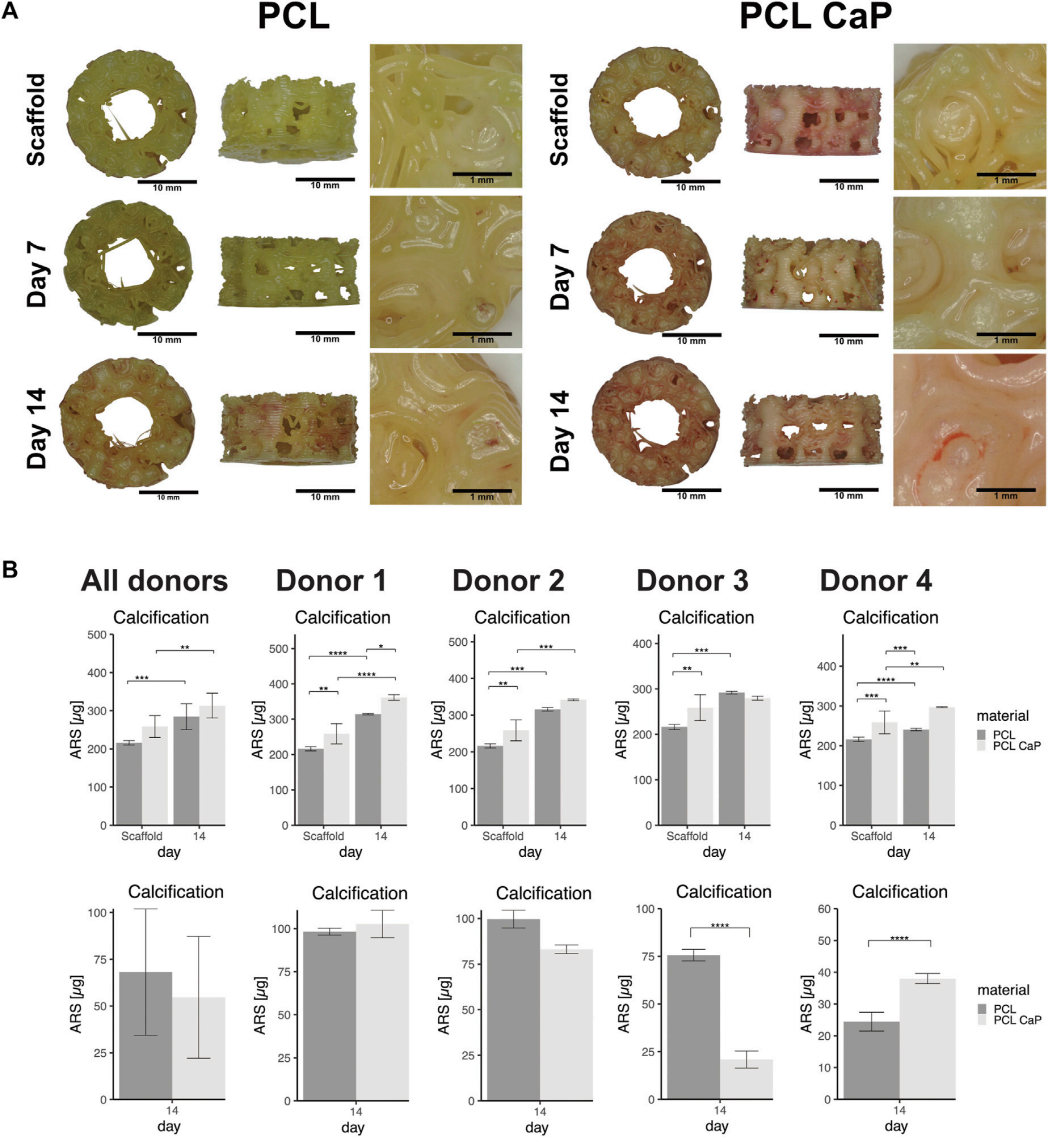
The calcification in MSC seeded scaffolds using Alizarin Red staining. **(A)** Light microscopy and morphological appearance of calcified areas over culture time for PCL and PCL-CaP. **(B)** Quantification of calcification in hMSC-seeded scaffolds on day 14 for different donors. Upper graphs: Total values for calcification from scaffolds without cells and hMSC-seeded constructs in average or depicted for individual donors. Lower graphs: Cell-based calcification (scaffold values subtracted) in hMSC-seeded constructs in average or depicted for individual donors. The statistical evaluation was performed using ANOVA with posthoc Tukey with *n* = 4 individual donors for each group. The samples were measured in technical duplicates.

For hMSC-seeded constructs (Figure 9A) an increase in calcium deposition with time of culture for both PCL and PCL-CaP was observed in the images. Morphologically, the best calcification was found in the PCL-CaP scaffolds on day 14, indicating a beneficial influence of this material composition on hMSC differentiation. The quantitative analyses indicated tentatively higher levels of calcification for PCL-CaP when background values from the scaffolds were not subtracted. After the subtraction of these values, the results indicated a high donor-donor variation (Figure 9B), and the effect was less consistent compared to the results from the MG63 cells.

## 4 Discussion

In this study, we developed 3D-printed osteoinductive bone scaffolds based on PCL and PCL combined with CaP. For the extrusion-based 3D printing process, an upscaled and simplified structure of the spongiosa with an interconnected porous structure was developed to print the scaffolds. The impact of the integrated calcium phosphate in the resulting scaffolds on biocompatibility and differentiation was investigated using cell and molecular biological evaluation methods and revealed an osteoinductive effect of the PCL-CaP scaffolds. The compression strength was not significantly influenced by the material composition in this study but resulted in comparable values to natural bone tissue.

For the 3D model, a pore size of about 2 mm with varying interconnectivities and an irregular distribution was chosen to create a simplified version of the natural structure in cancellous bone. The natural spongiosa in the bone has an average pore diameter of 300–600 μm (Lee et al., 2012), which is distinctly smaller than the pores in the developed porous model. For bone tissue engineering applications, generally, pore sizes of 200–600 µm are suggested in order to support bone growth and infiltration of blood vessels (Lim et al., 2010; Yang et al., 2021). An interconnected porous network enables the invasion of cells into the inner parts of the implant along with the supply of nutrients, oxygen, and biochemical signals. Thus, the interconnectivity is an important prerequisite for ensuring metabolic processes, cellular viability, and tissue regeneration throughout the implant (Kalfas, 2001; Kellner et al., 2002; Malda et al., 2007; Volkmer et al., 2008). Compared to the irregular pore size and distribution in the human bone spongiosa, the pores of the 3D model are round spheres, revealing only minor variations in their diameter. In addition, minor variations in the pore size or scaffold structure in this study may derive from the printing process itself, creating potential micropores.

The calculated surface area of the spongiosa-inspired 3D model includes only macropores. It can be assumed, that the surface area of the printed scaffold is actually larger due to micropores and gaps that might emerge between the individual layers during the printing process.

Depending on the area, porosities of cancellous bone range from 40% to 95% changing in response to different stimuli, such as mechanical forces (Di Luca et al., 2016; Morgan et al., 2018). In this perspective, the 35% porosity of the spongiosa-inspired 3D model is comparable to the denser region of the bone spongiosa.

In comparable studies, mostly constructs with a low height and a simple internal structure, such as a rectilinear infill pattern, were printed (Gómez-Lizárraga et al., 2017; Güney et al., 2018; Ma et al., 2018; Oberdiek et al., 2021; Sparks et al., 2022). In contrast, only a few studies of more complex or biomimetic structural designs for bone constructs, respectively, printing constructs that are customized for the patient are reported. Herath et al., 2021 created a more natural structure by machining the 3D model with a voronoi mosaic. For printing, a medical-grade PCL hydroxyapatite composite was applied (Herath et al., 2021). In the study of Hatt et al., 2022 an interlocking system using individual building blocks was used. This enables the generation of patient-specific constructs through the assembly of building blocks in accordance with the varying sizes and needs of patients (Hatt et al., 2022).

In principle, the outer shape of the scaffolds from this study can be adapted to any external geometry of bone and thus can be individually adapted to bone defects in patients. The cancellous bone-inspired scaffolds from this study might thus be used for bone defects of a critical size in the longterm. However, this study is meant to establish the design, the material, and the printing process and to assess the impact of the material on cell functions. In the context of the materials used in this study, PCL has been implanted in the clinic as a bone graft substitute in initial studies (Goh et al., 2015; Kim et al., 2018; Kobbe et al., 2020a).

Both the design of the printed construct and the selection of the material are equally important to ensure the mechanical stability of the printed construct or implant. The mechanical properties assessed by compression strength tests in our study revealed similar compression strengths for the two tested materials (PCL: 10.79 MPa ± 4.12 MPa, PCL-CaP: 9.7 MPa ± 3.15 MPa). However, due to the rounded shape of the construct and its overall geometry, the 3-point bending test was technically not feasible using our experimental settings and equipment. This would only be feasible if we adapt the shape of the implants towards more elongated structures.

Cancellous bone has a compression strength between 0.1 MPa and 16 MPa (Gerhardt and Boccaccini, 2010; Diaz-Rodriguez et al., 2018; Morgan et al., 2018). Thus the printed bone implant is within the range of the compression strength in natural bone. In this study, the compression strength was normalized to the mean area (83.075 mm^2^) which was calculated on the basis of the 3D model. However, due to the pore structure within the model, the surface area varies. This influences the compression strength values, when related to the maximum and minimum areas of the 3D model.

In the literature similar values for the mechanical properties of printed scaffolds have been reported. A Young’s modulus of 11.4 MPa for scaffolds with a voronoi structure using PLA and a porosity of 72.8% has been recently described (Herath et al., 2021).

Leemhuis et al. developed a mechano-hybrid-scaffold. This type of scaffold combines a collagen-based biomaterial containing aligned pores with a 3D printed PCL support structure, which revealed a stiffness of 9.56 MPa for the PCL-based part of the scaffold (Leemhuis, 2022). Porous scaffolds made of 80% PCL and 20% hydroxyapatite composite revealed an ultimate compressive strength of 3.7 MPa ±0.2 MPa (Lu et al., 2014).

Besides mechanical strength, biocompatibility is a crucial requirement for the application of a biomaterial in a medical environment (Mancha Sánchez et al., 2020).

To enhance the osteoinductive properties of the scaffolds, PCL was combined with CaP nanoparticles under heat, resulting in a composite material that was printable with the chosen extrusion-based printing technology. Using this method, a maximum of 30% (w/w) could be integrated into the composite. For the study, however, a ratio of 20% CaP (w/w) was used. Besides using heat to blend PCL with CaP, solving PCL using chloroform may enable higher rates of CaP in the composite. However, the printability and printing accuracy of the material were reported to decrease when using the solvent (Zimmerling et al., 2021a). CaP and hydroxyapatite are closely related compounds. Hydroxyapatite is often used as a biomaterial for bone grafts, implants, and tissue-engineered scaffolds due to its excellent biocompatibility and its ability to support bone regeneration (Lei et al., 2015; Combes et al., 2016; Kolanthai et al., 2016; Terukina et al., 2017). CaP in general exists in various forms, while hydroxyapatite is a specific form of CaP with a unique crystal structure found in the mineral phase of natural bone (Tay et al., 1999; Kim et al., 2018; Yu and Wei, 2021; Chen et al., 2023). CaP based materials can release calcium and phosphate ions, which are essential for the differentiation of hMSC into osteoblasts and thus promote bone formation (Tavares et al., 2020). These ions are crucial for the bone mineralization process and the commitment of hMSC towards the osteoblast lineage. hMSC-derived osteoblasts deposit calcium and phosphate ions, which crystallize and form hydroxyapatite-like mineral structures. Some studies have suggested that amorphous CaP exhibits enhanced osteoinductive properties compared to hydroxyapatite (McGann et al., 1983; Hu et al., 2007; Dorozhkin, 2021). Further, CaP seem to have a higher solubility compared to hydroxyapatite (Akkineni et al., 2015) thus providing the option to trigger bone differentiation by the release of Ca ions.

Therefore, we analyzed the impact of the printed scaffold and the material composition on cell performance. For initial studies, MG63 cells were used due to their lower heterogeneity in terms of cellular composition and donor-donor variation (Phinney, 2012; Costa et al., 2021). In addition, MG63 cells are more robust than hMSC. After the colonization with MG63 cells showed promising results, the scaffolds were also seeded with hMSC to assess the impact of bone implants with patient-derived cells.

By assessing the DNA amount after seeding (day 1), the early cell adhesion or seeding efficacy for MG63 cells was found to be similar for both types of materials. For the PCL-based constructs, the significant increase in the DNA amount indicates cell proliferation over the first 7 days. However, no proliferation was observed for cells grown on the scaffolds with calcium phosphate. This might be due to the induction of osteogenic differentiation, which is often associated with slowing down cellular proliferation processes (Golub and Boesze-Battaglia, 2007; Sharma et al., 2014; Rutkovskiy et al., 2016; Ma et al., 2018). However, besides the lower DNA amounts, the adhesion molecule integrin ß1 on day 7 was less expressed in the MG63 cells grown on the CaP scaffolds but increased again with time. In conjunction, this lower integrin expression and the lower DNA amounts may suggest that the cell material interaction or cell performance on the PCL-CaP in the earlier phases of culture is slightly reduced compared to PCL. In agreement with these observations, the cytotoxicity determined by LDH quantification from the supernatant of cell-seeded constructs revealed significantly higher cytotoxicity for PCL-CaP samples than for PCL alone after 24 h (Supplementary Figure S3). This effect seems to diminish with time of culture for the PCL-CaP scaffolds. However, it remains unclear if, for instance, free CaP nanoparticles interfere with the cell function or viability.

On the morphological level, the CLSM and SEM data approved that cells on the material were vital by showing a typical elongated phenotype and by covering the surface of both types of materials.

The osteoinductive effect of PCL and PCL-CaP scaffolds on MG63 cells was investigated by various methods.

For MG63 cells, the data from different methods, such as PCR, ALP, and ARS, and several osteogenic differentiation markers, underline an overall osteoinductive effect of the PCL-CaP scaffolds. For this type of material, a significant increase in the cell-mediated calcification process (background of scaffolds subtracted) over culture time was observed, along with higher levels in the gene expression of osteogenic differentiation-related markers such as osteocalcin and collagen type 1.

In parallel, a decrease in ALP activity over time for both tested materials was observed. This decrease in ALP is often associated with the initiation of the mineralization process (Rutkovskiy et al., 2016; Ma et al., 2018) in osteogenesis, characterized by the deposition of calcium and phosphate minerals to form hydroxyapatite, the main mineral component of bone (Golub and Boesze-Battaglia, 2007; Sharma et al., 2014).

For hMSC, the calcification process was investigated using ARS and quantification for several donors of hMSC. On the morphological level, the same trend as for the MG63 cells was observed in the ARS. The hMSC on the PCL-CaP indicated the highest temporal increase in calcification. Although hMSC grown on the PCL with CaP showed the strongest signal in the images and in the total values for calcification within the constructs, the results differed between individual donors and were not consistent when the values from the scaffolds were subtracted. Although donor-to-donor variation may play a major role in this observation, complete extraction of the AR from the 3D scaffolds followed by photometric evaluation is a critical step. Thus, influences on the data by the extraction process itself or by dynamic or individual changes of calcium levels within the scaffold cannot be excluded. In addition, the differentiation between the material and cell-derived contributions to the calcification process remains a critical issue for quantification by AR. Despite these critical factors, ARS and quantification for the non-seeded scaffolds show that at least parts of the CaP particles are freely accessible on the surface of the composite scaffolds. CaP-containing scaffolds in combination with the cells (CaP from scaffolds and cells in total) indicated the highest calcification for both MG63 cells and hMSC. This indicates the osteoinductive potential of the printed PCL-CaP constructs.

According to the literature, complete degradation of PCL can take as long as 3–4 years, or 1–2 years depending on the design of the implant or the molecular weight of the PCL (Bliley et al., 2015; Arakawa et al., 2017). We assume that the CaP nanoparticles lead to changes in the degradation process. However, the time frame would also depend on the physiological conditions *in vivo*, including biomechanical aspects. To address the degradation in a relevant biomedical context, *in vivo* studies would be necessary, which are beyond the scope of the present study.

## 5 Conclusion

In this study, we designed a bone spongiosa-inspired model for the extrusion-based 3D printing of an osteoinductive implant. The scaffolds were printed using PCL as reference material and PCL-CaP to enhance the osteoinductive properties of the implant. The printed scaffolds revealed a suitable compression strength, which was not impaired by CaP. The composite PCL-CaP-printed constructs further revealed a beneficial effect on the osteogenic differentiation in the cell line MG63 and achieved highest levels in terms of the total calcium content in constructs using hMSC, thus underlining the osteoinductive properties.

## Data Availability

The original contributions presented in the study are included in the article/Supplementary Material, further inquiries can be directed to the corresponding author.
