# Supplementary material for: Extrusion-based 3D printing of osteoinductive scaffolds with a spongiosa-inspired structure
Source: Front Bioeng Biotechnol. 2023 Sep 18;11:1268049. doi: 10.3389/fbioe.2023.1268049 (PMC10544914; doi:10.3389/fbioe.2023.1268049)
Supplement: Supplementary file 1 [file DataSheet1.docx]

Supplementary Material

Extrusion-based 3D printing of osteoinductive scaffolds with a spongiosa-inspired structure

Julie Kühl^1^, Stanislav Gorb^2^, Matthias Kern^3^, Tim Klüter^1^, Sebastian Kühl^4^, Andreas Seekamp^1^, Sabine Fuchs^1*^

^1^ Experimental Trauma Surgery, Department of Orthopedics and Trauma Surgery, University Medical Center, Kiel, Germany

^2^ Department of Functional Morphology and Biomechanics, Kiel University, Kiel, Germany

^3^ Department of Prosthodontics, Propaedeutics and Dental Material, University Medical Center, Kiel, Germany

^4^ Chair of Communications, Department of Electrical and Information Engineering, Kiel University, Kiel, Germany

*** Correspondence:**Sabine Fuchs
[sabine.fuchs@uksh.de](mailto:sabine.fuchs@uksh.de)

# Supplementary Data

## Python script of the 3D model

from sdf import *

f = rounded_cylinder(10, 0.0, 10) - rounded_cylinder(4, 0.0, 12)

# Grid with normal distributed offset

sphere_distance = 4

erodation_radius = 0.1

shift = False

# Layers

for z in np.arange(-7, 7, sphere_distance):

shift = not shift

# Grid

for x in np.arange(-12, 12, sphere_distance):

for y in np.arange(-12, 12, sphere_distance):

# Normal distributed offset mean: 2, std: 0.2

offset = np.random.normal(0, 0.2, size=3)

coords = np.array([x,y,z])

# Shift every 2. layer

if shift:

coords[0] += 1

coords[1] += 1

# Normal distributed radius mean: 2, std: 0.2

radius = np.random.normal(2, 0.2)

f = f - sphere(radius).translate(coords+offset)

f = f.erode(erodation_radius)

f.save('in.stl')

# Supplementary Figures and Tables

## Supplementary Figures

**
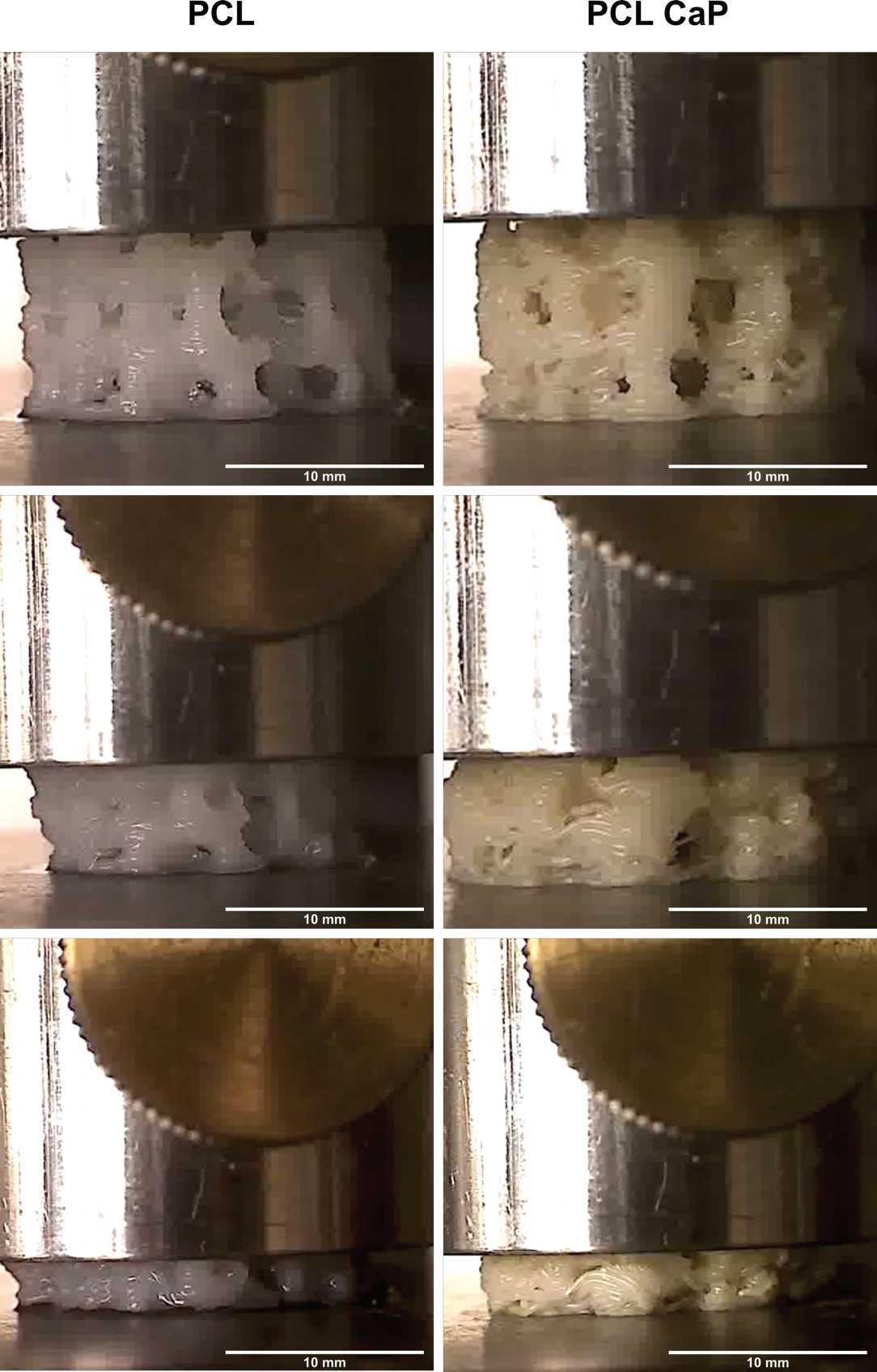
**

**Supplemental Figure 1: Illustration of compression strength test for printed scaffolds made of PCL and PCL-CaP at different stages of compression.** The scaffolds were aligned as shown in the illustrations. For each group, 12 samples (n=12) were compressed.


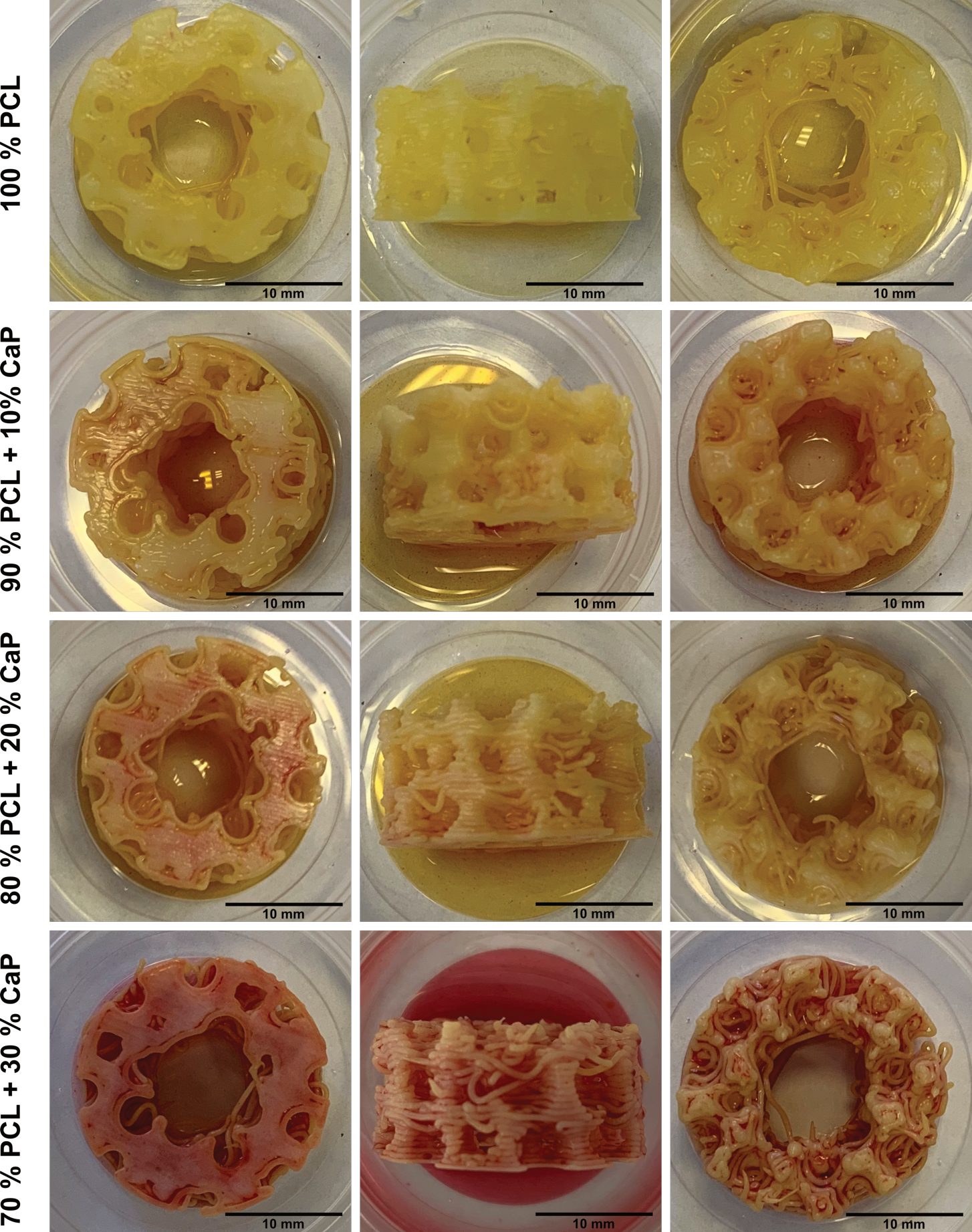


**Supplemental Figure 2: Alizarin Red Staining of cell-free scaffolds with different CaP concentrations.** The scaffold of PCL without CaP serves as control.


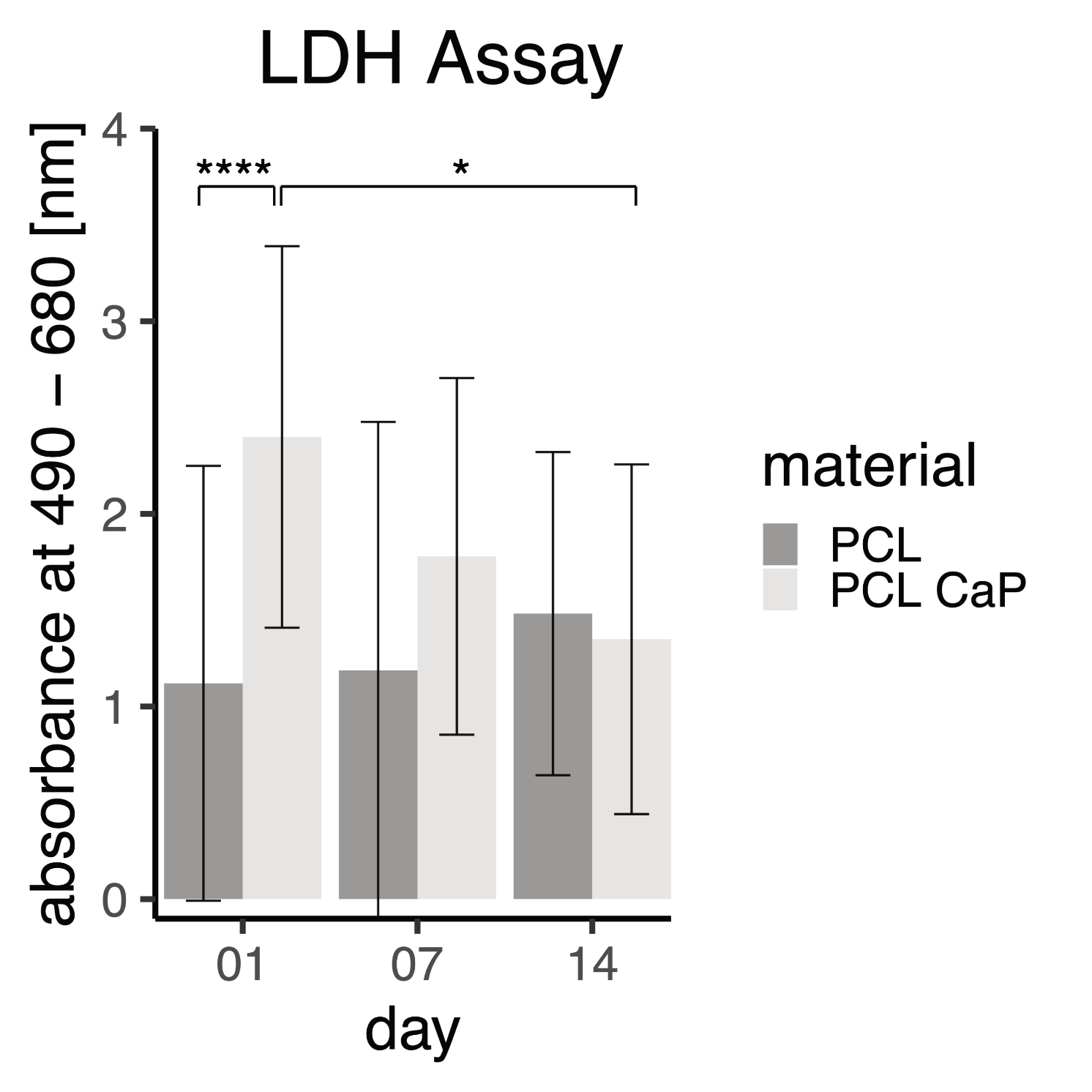
**Supplemental Figure 3: Lactate dehydrogenase assay of the supernatants of the MG63 cell-seeded scaffolds.** LDH release for MG63 cells seeded on scaffolds consisting of PCL and PCL-CaP over the time. The statistical evaluation was performed using ANOVA with posthoc Tukey with n = 6, from six different passages with three technical replicates. Statistical significance level: p < 0.05 (*), p < 0.001 (***), and p < 0.0001 (****).
